# TIBC, PLR, and EBV DNA load correlate with hepatic injury in EBV-associated infectious mononucleosis

**DOI:** 10.3389/fped.2026.1727430

**Published:** 2026-04-15

**Authors:** Shengfeng Sun, Mingqi Wang, Shuyan Yang, Yuanyuan Huang

**Affiliations:** Department of Outpatient and Emergency, Children's Medical Center, The First Hospital of Jilin University, Changchun, China

**Keywords:** hepatic injury, human herpesvirus, infectious mononucleosis, iron metabolism, pediatric infectious diseases

## Abstract

**Objective:**

To analyze clinical features of hepatic injury in children with EBV-associated infectious mononucleosis (EBV-IM) and identify its risk factors.

**Methods:**

A retrospective analysis of the clinical data of children with EBV-IM admitted to the First Hospital of Jilin University from March 2023 to March 2025 was conducted. Confounding factors were balanced by propensity score matching (PSM). The children were divided into a hepatic injury group and a non-hepatic injury group. The risk factors were examined using binary logistic regression. Furthermore, the diagnostic performance of these risk factors was assessed and presented using receiver operating characteristic (ROC) curves.

**Results:**

After PSM, 52 pairs of patients were matched. Statistically significant differences in white blood cell count, the proportion of neutrophils, the proportion and absolute value of lymphocytes, the platelet–to-lymphocyte ratio (PLR), hepatosplenomegaly, whole blood EBV DNA load >1 × 10^5^ copies/mL, and total iron-binding capacity(TIBC) were detected(all *P* < 0.05). Multivariate logistic regression analysis revealed that TIBC and whole blood EBV DNA load >1 × 10^5^ copies/mL were risk factors, whereas the PLR was a protective factor. ROC curve analysis showed that the cutoff values of TIBC and reciprocal platelet-to-lymphocyte ratio (rPLR) for the diagnosis of EBV-IM-related hepatic injury were 53.250 μmol/L and 0.052, with areas under the curve of 0.652 and 0.713, sensitivities of 69.2% and 53.8%, and specificities of 62.7% and 88.2%, and these two indicators could be used as diagnostic markers for hepatic injury in children with EBV-IM.

**Conclusion:**

EBV-IM-related hepatic injury in children correlates with iron metabolism. TIBC and whole blood EBV DNA load (>1 × 10^5^ copies/mL) are independent risk factors, whereas the PLR is a protective factor.

## 1 Introduction

EBV-IM is an acute self-limiting infectious disease that is often caused by Epstein–Barr virus (EBV) infection. It can occur at any age, with fever, pharyngitis, and cervical lymphadenopathy as the main manifestations. Although EBV-IM is mostly self-limiting, epidemiological data show that the number of pediatric EBV-IM cases has increased annually in recent years (1–3). Clinically, some children can also have multiple organs and systems involved, resulting in functional damage to these organs and systems, among which hepatic injury is the most common. In mild cases, children may have no obvious symptoms or present with self-limiting jaundice hepatitis; in severe cases, they may progress to EBV-associated hemophagocytic syndrome or develop liver failure, which is life-threatening (4). This study retrospectively analyzed the clinical data of hospitalized children diagnosed with EBV-IM at the First Hospital of Jilin University from March 2023 to March 2025, with systematic statistical analysis performed on the data including general data, clinical symptoms, routine blood tests, inflammatory indicators, EBV DNA load, iron metabolism indicators and other relevant clinical indicators, aiming to clarify the clinical characteristics of children with EBV-IM-related hepatic injury, screen and identify the risk factors for EBV-IM-related hepatic injury, and ultimately reduce the incidence of severe hepatic injury and optimize the clinical prognosis of affected children.

## 2 Materials and methods

### 2.1 Study design and ethical approval

A retrospective analysis of the clinical data of children who were diagnosed with EBV-IM and admitted to the First Hospital of Jilin University from March 2023 to March 2025, including general information, symptoms and signs, routine blood tests, high-sensitivity C-reactive protein(CRP), whole blood EBV DNA load, biochemistry, iron metabolism, etc., was performed. This study was approved by the Medical Ethics Committee of the First Hospital of Jilin University (No. 2025-406).

### 2.2 Diagnostic criteria for EBV-IM

The diagnostic criteria for EBV-IM were adopted from the Expert Consensus on Diagnostic and Therapeutic Principles for EBV Infection-Related Diseases in Children (2021 Edition) (5). Specifically, clinical diagnostic cases met any 3 of the following clinical manifestations plus any 1 nonspecific laboratory finding; confirmed cases met any 3 of the following clinical manifestations plus any 1 laboratory evidence of primary EBV infection.

Clinical manifestations: (1) Fever. (2) Pharyngitis. (3) Cervical lymphadenopathy. (4) Hepatomegaly. (5) Splenomegaly. (6) Palpebral edema.

Laboratory evidence of primary EBV infection: (1) Positive for anti-EBV-CA-IgM and anti-EBV-CA-IgG antibodies, with negative EBV-NA-IgG. (2) Positive for anti-EBV-CA-IgG antibodies, with low avidity.

Nonspecific laboratory findings: (1) Peripheral blood atypical lymphocyte ratio ≥0.10. (2) For children over 6 years old, the peripheral blood lymphocyte ratio >0.50 or absolute lymphocyte count >5.0 × 10^9^/L.

### 2.3 Inclusion and exclusion criteria

The inclusion criteria were as follows: (1) The patients met the diagnostic criteria for EBV-IM with a first diagnosis. (2) The patients had complete clinical and laboratory data.

Exclusion criteria: (1) The comorbidities of other known diseases could cause hepatic injury, such as viral hepatitis (hepatitis A, B, C, D, E, etc.), autoimmune liver disease, genetic metabolic liver disease, etc. (2) Severe congenital diseases, immunodeficiency diseases, malignant tumors, etc. (3) Long-term use of immunosuppressants or glucocorticoids. (4) Confirmed co-infections with other pathogens, such as cytomegalovirus, Streptococcus, or Mycoplasma pneumoniae.

### 2.4 Grouping criteria for hepatic injury

The children were divided into a hepatic injury group and a non-hepatic injury group according to the presence or absence of hepatic injury. Based on relevant literature, the diagnostic criteria for hepatic injury were as follows: total bilirubin ≥4 mg/dL (68 μmol/L) or alanine aminotransferase (ALT) exceeding twice the age-appropriate upper limit of normal, with no administration of hepatic function-affecting drugs within 1 month prior to the visit (6).

### 2.5 Clinical assessment of hepatosplenomegaly

The recording of the status of hepatosplenomegaly in pediatric patients adopted a dual assessment of clinical palpation and abdominal ultrasound verification. Positive palpation for hepatomegaly was defined as follows: during quiet breathing, hepatomegaly (liver enlargement) of more than 3 cm could be palpated at the right costal margin in infants and young children, or the liver could be palpated below the right costal margin in children aged 4 years or older. Positive palpation for splenomegaly was defined as the edge of the spleen being palpable at the left costal margin (7). A positive result on palpation or a positive result on ultrasound was determined to indicate the presence of hepatomegaly or splenomegaly. When the two results were inconsistent, the ultrasound result prevailed.

### 2.6 Laboratory measurements

All relevant blood samples were obtained through the first venous blood collection from pediatric patients within 24 h after their admission to the hospital. All laboratory tests of the enrolled samples in this study were sent to the Department of Laboratory Medicine of the First Hospital of Jilin University for testing. Hematologic analysis was performed using the Sysmex XN-9000 automated hematology analyzer system (Sysmex Corporation, Kobe, Japan), following the manufacturer's standard protocols. Measurements of biochemical analytes were run with Ortho VITROS 5600 (Ortho-Clinical Diagnostics), a dry versatile chemistry system, according to the manufacturer's instructions. All laboratory procedures were performed by trained laboratory staff. The Laboratory was accredited to ISO 15189:2012 Medical Laboratories-Particular Requirements for Quality and Competence by China National Accreditation Service for Conformity Assessment in 2012. The entire workflow of all laboratory tests was strictly implemented in compliance with the ISO 15189 standard, and the test results had stable traceability, reliability, and clinical comparability. Reference ranges for relevant laboratory indicators were based on reference intervals for common clinical biochemical test items in children issued by the National Health Commission of the People's Republic of China on April 9, 2021.

### 2.7 Propensity score matching (PSM)

Since the risk of Epstein–Barr virus (EBV) infection may increase with age, propensity score matching (PSM) was performed to select age- and gender-matched cases between the two groups. This procedure was implemented to balance the baseline confounding variables (age and gender) between the hepatic injury and non-hepatic injury groups. Propensity scores were estimated using a multivariable logistic regression model, with hepatic injury status as the dependent variable, and age (as a continuous variable) and gender as independent variables. Subsequently, 1:1 nearest-neighbor matching was conducted without replacement, using a caliper width of 0.1 standard deviations of the logit-transformed propensity score. Standardized mean difference (SMD) was calculated to evaluate covariate balance after matching, with an SMD <0.1 defined as adequate balance.

### 2.8 Statistical analysis

Statistical analyses were performed via R 4.2 and SPSS 26.0 software. Categorical data were expressed as frequencies and percentages; continuous data with a normal distribution were expressed as the mean ± standard deviation, and those with a non-normal distribution were expressed as M (P25, P75). The chi-square test was used for comparisons of categorical data; the independent samples t test was used for continuous data with a normal distribution, and nonparametric tests were used for those with a non-normal distribution. Multivariate logistic regression analysis was performed for univariate factors with statistically significant differences. Receiver operating characteristic (ROC) curves were plotted to analyze the diagnostic performance of each factor. A two-sided *P* < 0.05 was considered to indicate statistical significance.

## 3 Results

### 3.1 Comparison of general data and hospitalization season between the two groups

**Figure 1:**
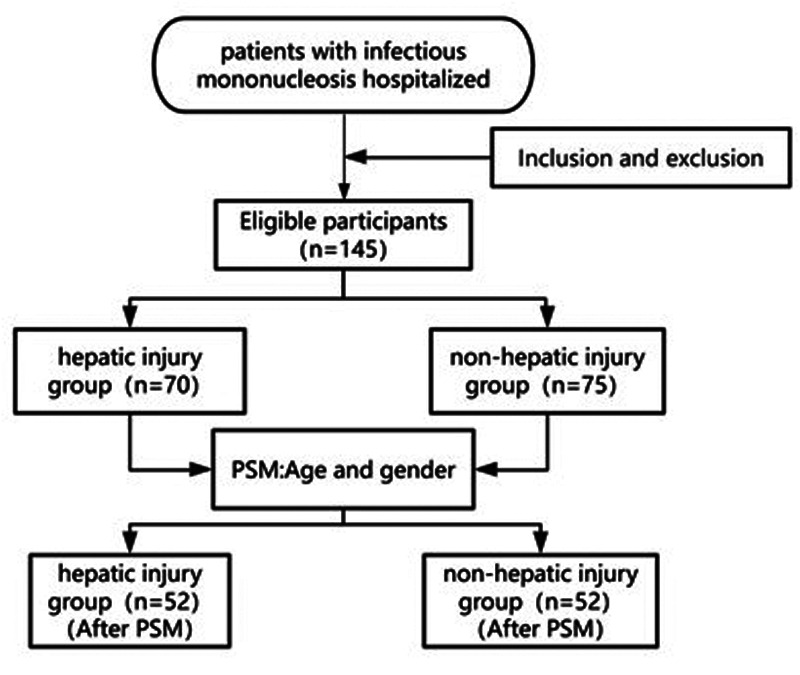
Participant selection flowchart. Initially, hospitalized patients with EBV-IM were evaluated against predefined inclusion and exclusion criteria, yielding 145 eligible participants. These participants were divided into a hepatic injury group (*n* = 70) and a non-hepatic injury group (*n* = 75). Subsequently, 1:1 PSM based on age and gender was performed, resulting in 52 patients in each group after matching.

Before PSM, a total of 145 children with EBV-IM admitted to the First Hospital of Jilin University from March 2023 to March 2025 were enrolled, including 70 patients (48.28%) in the hepatic injury group and 75 patients (51.72%) in the non-hepatic injury group. The patients in the hepatic injury group were significantly older than those in the non-hepatic injury group (*P* < 0.05). The hepatic injury group included 43 males and 27 females aged 1–16 years, with a mean age of 7.53 ± 3.02 years. In the non-hepatic injury group, 37 males and 38 females aged 2–15 years were included, with a mean age of 6.13 ± 2.98 years (as shown in Table 1). After PSM to adjust for age and gender, 52 pairs of patients were successfully matched. The flowchart of patient selection in this study is shown in Figure 1. In the hepatic injury group (52 patients), there were 28 males and 24 females aged 1–15 years, with a median age of 7 years. In the non-hepatic injury group (52 patients), there were 26 males and 26 females aged 3–15 years, with a median age of 7 years. After PSM, no differences in age or gender were observed between the two groups (*P* > 0.05) (as shown in Table 2).

**Table 1:** Comparison of general data and hospitalization season between the two groups before PSM.

Item	Hepatic injury group (*n* = 70)	Non-hepatic injury group (*n* = 75)	t/z/*χ*^2^	*P*
Age (years)	7.53 ± 3.02	6.13 ± 2.98	−2.80	0.006
Gender, *n* (%)			2.14	0.143
Male	43 (61.43)	37 (49.33)		
Female	27 (38.57)	38 (50.67)		
BMI (kg/m²)	16.13 ± 2.94	15.44 ± 2.48	−1.55	0.124
Season, *n* (%)			5.15	0.161
Spring	12 (17.14)	16 (21.33)		
Summer	30 (42.86)	25 (33.33)		
Autumn	20 (28.57)	16 (21.33)		
Winter	8 (11.43)	18 (24.00)		

**Table 2:** Comparison of general data and hospitalization season between the two groups after PSM.

Item	Hepatic injury group (*n* = 52)	Non-hepatic injury group (*n* = 52)	t/z/χ^2^	*P*
Age (years)	7.00 (5.00, 8.00)	7.00 (5.75, 8.00)	−0.86	0.39
Gender, *n* (%)			0.15	0.69
Male	28 (53.85)	26 (50.00)		
Female	24 (46.15)	26 (50.00)		
BMI (kg/m²)	14.75 (13.66, 16.24)	15.24 (14.31, 17.00)	−1.33	0.19
Season, *n* (%)			3.57	0.31
Spring	12 (23.08)	12 (23.08)		
Summer	16 (30.77)	22 (42.31)		
Autumn	11 (21.15)	12 (23.08)		
Winter	13 (25.00)	6 (11.54)		

### 3.2 Comparison of length of hospital stay and clinical symptoms between the two groups after PSM

No statistically significant differences were observed between the two groups in terms of length of hospital stay, fever, duration of fever, peak temperature, pharyngitis, cervical lymphadenopathy, maximum diameter of the cervical lymph nodes, hepatomegaly, splenomegaly, hepatomegaly or splenomegaly, or palpebral edema (all *P* > 0.05). However, a statistically significant difference was noted in the rates of hepatosplenomegaly between the two groups (*P* < 0.05). The details are shown in Table 3.

**Table 3:** Comparison of length of hospital stay and clinical symptoms between the two groups after PSM.

Item	Hepatic injury group (*n* = 52)	Non-hepatic injury group (*n* = 52)	t/z/χ^2^	*P*
Length of hospital stay (d)	6.50 (5.00, 8.25)	6.00 (4.75, 8.00)	−0.49	0.63
Fever, *n* (%)			0.27	0.60
Absent	10 (19.23)	8 (15.38)		
Present	42 (80.77)	44 (84.62)		
Duration of fever (d)	5.00 (2.75, 8.25)	5.50 (4.00, 7.25)	−0.73	0.46
Peak temperature (℃)	38.65 (38.15, 39.20)	38.90 (38.30, 39.23)	−0.85	0.39
Pharyngitis, *n* (%)			0.00	1.00
Absent	7 (13.46)	7 (13.46)		
Present	45 (86.54)	45 (86.54)		
Maximum diameter of cervical lymph nodes (cm)	2.00 (1.50, 2.50)	1.75 (1.50, 2.50)	−1.49	0.14
Hepatomegaly, *n* (%)			0.87	0.35
Absent	38 (73.08)	42 (80.77)		
Present	14 (26.92)	10 (19.23)		
Splenomegaly, *n* (%)			0.15	0.69
Absent	25 (48.08)	27 (51.92)		
Present	27 (51.92)	25 (48.08)		
Hepatosplenomegaly, *n* (%)			4.30	0.04
Absent	39 (75.00)	47 (90.38)		
Present	13 (25.00)	5 (9.62)		
Neither hepatomegaly nor splenomegaly, *n* (%)			0.16	0.69
Absent	28 (53.85)	30 (57.69)		
Present	24 (46.15)	22 (42.31)		
Palpebral edema, *n* (%)			0.39	0.53
Absent	19 (36.54)	16 (30.77)		
Present	33 (63.46)	36 (69.23)		

### 3.3 Comparison of routine blood test and inflammatory indicators between the two groups after PSM

There were no significant differences in the absolute neutrophil count, red blood cell count, mean corpuscular volume (MCV), hemoglobin, platelet count, mean platelet volume (MPV), MPV-to-platelet ratio (MPV/PLT), CRP, or procalcitonin between the two groups (*P* > 0.05); significant differences in white blood cell count, the neutrophil percentage, the lymphocyte percentage, the absolute lymphocyte count, the neutrophil‒lymphocyte ratio (NLR), or the platelet-to‒lymphocyte ratio (PLR) were detected between the two groups (*P* < 0.05). The details are shown in Table 4.

**Table 4:** Comparison of routine blood tests and inflammatory indicators between the two groups after PSM.

Item	Hepatic injury group (*n* = 52)	Non-hepatic injury group (*n* = 52)	t/z/χ^2^	*P*
White blood cell count (10^9^/L)	14.36 (11.67, 19.83)	11.14 (8.89, 15.71)	−3.32	<0.01
Neutrophil proportion (ratio)	0.17 (0.14, 0.21)	0.23 (0.18, 0.27)	−3.55	<0.01
Lymphocyte proportion (ratio)	0.76 (0.69, 0.81)	0.70 (0.64, 0.76)	−2.84	<0.01
Absolute neutrophil count (10^9^/L)	2.42 (1.89, 3.17)	2.87 (1.80, 3.64)	−0.80	0.42
Absolute lymphocyte count (10^9^/L)	10.81 (7.75, 15.12)	7.35 (5.67, 10.22)	−3.86	<0.01
Neutrophil-to-lymphocyte ratio (NLR)	0.23 (0.16, 0.32)	0.35 (0.23, 0.44)	−3.59	<0.01
Red blood cell count (10^12^/L)	4.48 ± 0.35	4.41 ± 0.30	−1.14	0.26
Mean corpuscular volume (fL)	82.18 ± 3.29	81.91 ± 2.76	−0.25	0.81
Hemoglobin (g/L)	123.00 (117.75, 127.25)	123.00 (115.50, 128.25)	−0.40	0.69
Platelet count (10^9^/L)	213.50 (177.25, 265.25)	221.00 (189.50, 255.25)	−0.46	0.64
Mean platelet volume (MPV) (fL)	9.40 (9.00, 9.70)	9.30 (8.80, 9.55)	−0.66	0.51
Platelet-to-lymphocyte ratio (PLR)	18.64 (14.38, 29.12)	27.42 (22.66, 38.00)	−3.53	<0.01
MPV-to-platelet ratio (MPV/PLT)	4.57 (3.82, 5.30)	3.98 (3.36, 4.48)	−1.62	0.11
CRP (mg/L)	4.41 (1.92, 6.33)	5.73 (1.87, 14.79)	−1.35	0.18
Procalcitonin (ng/mL)	0.16 (0.10, 0.21)	0.17 (0.14, 0.23)	−0.66	0.51

### 3.4 Comparison of iron metabolism indicators and EBV DNA load between the two groups after PSM

There were no significant differences in iron or ferritin levels between the two groups (*P* > 0.05); the TIBC in the hepatic injury group was significantly greater than that in the non-hepatic injury group (*P* < 0.05). To explore the correlation between whole blood EBV DNA load and the development of hepatic injury in EBV-IM children, the EBV DNA load was dichotomized at its median value into high-load (>1 × 10^5^ copies/mL) and low-load (≤1 × 10^5^ copies/mL) groups. Stratified analysis revealed that the proportion of patients with high EBV DNA load was significantly higher in the hepatic injury group (61.54%) than in the non-hepatic injury group (34.62%). A chi-square test confirmed a statistically significant difference in EBV DNA load distribution between the two groups (*χ*^²^ = 7.55, *P* < 0.01). The details are shown in Table 5.

**Table 5:** Comparison of iron metabolism indicators and EBV DNA load between the two groups after PSM.

Item	Hepatic injury group (*n* = 52)	Non-hepatic injury group (*n* = 52)	t/z/χ^2^	*P*
Iron (μmol/L)	9.35 (7.28, 12.72)	9.20 (6.30, 10.95)	−1.34	0.18
Ferritin (μg/L)	87.95 (54.92, 140.75)	85.80 (64.30, 129.75)	−0.08	0.94
TIBC (μmol/L)	55.75 (50.77, 61.05)	51.00 (48.00, 57.00)	−2.66	<0.01
EBV DNA load > 1 × 10⁵ copies/mL, *n* (%)			7.55	<0.01
Absent	20 (38.46)	34 (65.38)		
Present	32 (61.54)	18 (34.62)		

### 3.5 Multivariate logistic analysis of EBV-IM-related hepatic injury

Variables with statistically significant differences in the univariate analysis were included in the multivariate logistic regression analysis. The results revealed that TIBC and EBV DNA load exceeding 1 × 10^5^ copies/mL were risk factors for EBV-IM-related hepatic injury, whereas the PLR was a protective factor. The details are shown in Table 6.

**Table 6:** Multivariate logistic analysis of EBV-IM-related hepatic injury.

Risk factor	*β*	S.E.	Wald χ^2^	OR	95% CI	*P*
TIBC	0.107	0.034	9.825	1.113	1.041–1.191	0.002
EBV DNA load >1 × 10⁵ copies/mL	1.381	0.499	7.672	3.980	1.498–10.575	0.006
PLR	−0.062	0.022	8.141	0.940	0.900–0.981	0.004

In the subsequent receiver operating characteristic (ROC) curve analysis, given that PLR was an independent protective factor against hepatic injury in children with EBV-IM, the reciprocal of PLR was calculated to construct the reciprocal platelet-to-lymphocyte ratio (rPLR) in this study. This conversion was performed to unify the risk interpretation direction of the indicators and allow direct and intuitive comparison of the diagnostic efficacy of different indicators on the same dimension. After conversion, rPLR acted as a risk factor for hepatic injury, which was completely consistent with the direction of the risk effect of TIBC.

**Figure 2:**
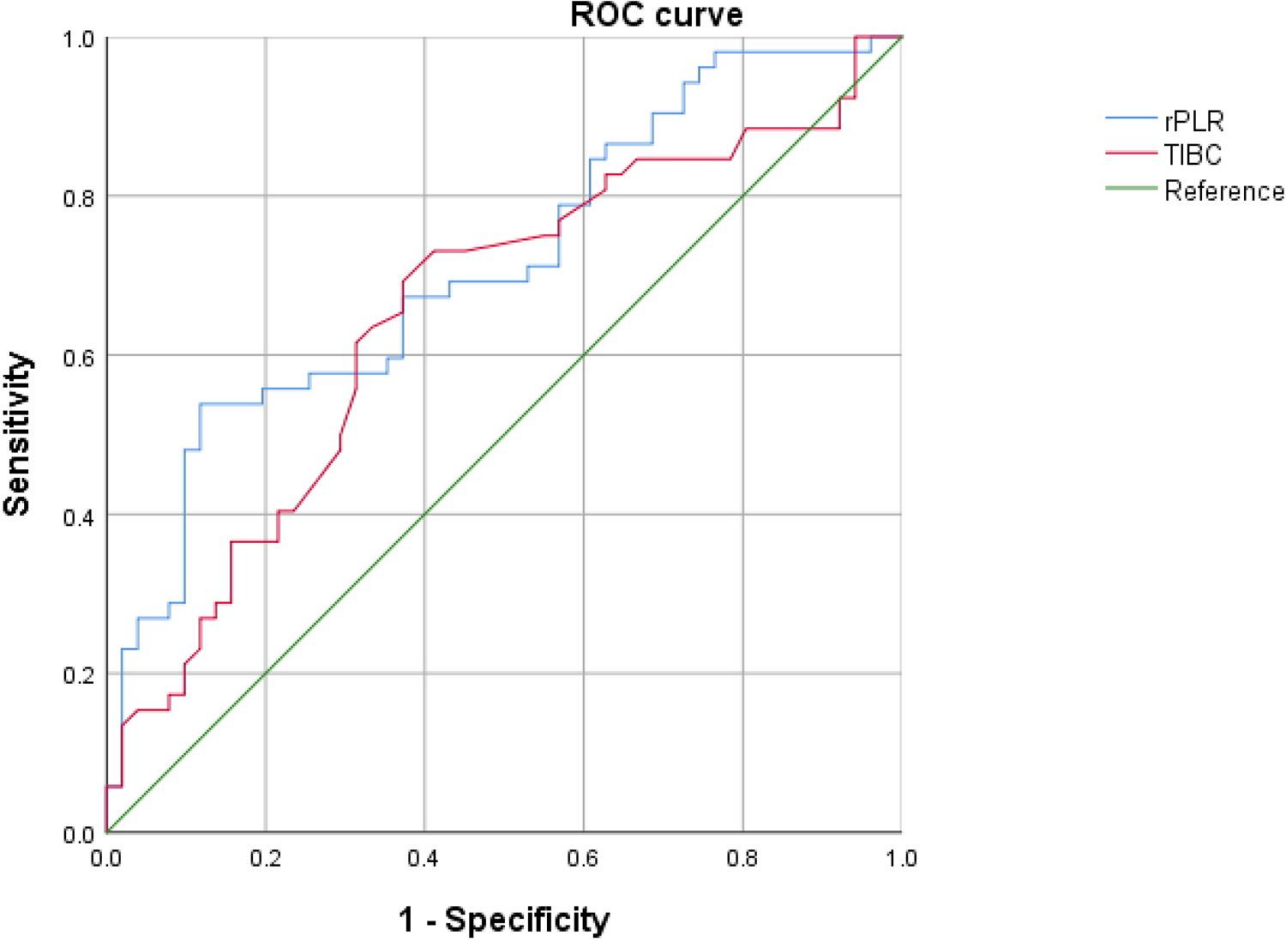
ROC curves of rPLR and TIBC for the diagnosis of EBV-IM-related hepatic injury in children. Receiver operating characteristic (ROC) curves were constructed to evaluate the diagnostic efficacy of the reciprocal platelet-to-lymphocyte ratio (rPLR) and total iron-binding capacity (TIBC) for hepatic injury in children with EBV-IM. The green diagonal line represents the reference line [area under the curve (AUC) = 0.5]. Detailed diagnostic performance parameters, including cutoff values, AUCs, sensitivities, specificities, and Youden indices, are presented in Table 7.

The final ROC curve analysis indicated that both TIBC and rPLR exhibited favorable diagnostic efficacy and could serve as effective diagnostic indicators for concurrent hepatic injury in children with EBV-IM, with certain auxiliary clinical screening value. Specifically, the cutoff values of TIBC and rPLR for the diagnosis of EBV-IM-related hepatic injury were 53.250 μmol/L and 0.052, with areas under the curves of 0.652 and 0.713, sensitivities of 69.2% and 53.8%, and specificities of 62.7% and 88.2%, respectively. Nevertheless, the extrapolated application efficacy of the above indicators still needs to be further verified by future multicenter, large-sample prospective clinical studies. Details are shown in Table 7 and Figure 2.

**Table 7:** Diagnostic value of TIBC and rPLR for hepatic injury in children with EBV-IM.

Item	AUC	S.E.	*P*	95% CI	Cutoff	Sensitivity	Specificity	Youden index
TIBC (μmol/L)	0.652	0.055	0.008	0.545–0.759	53.250	0.692	0.627	0.319
rPLR	0.713	0.051	<0.001	0.614–0.812	0.052	0.538	0.882	0.420

## 4 Discussion

EBV is the pathogen responsible for infectious mononucleosis. It is a human lymphocyte-tropic herpesvirus belonging to the gammaherpesvirus family. The spherical EBV particles are structurally composed, from the outside inward, of a viral envelope, tegument, and icosahedral capsid. Enclosed within the viral capsid is a linear double-stranded DNA genome approximately 172 kb in length, which encodes over 80 viral proteins, multiple noncoding RNAs (ncRNAs), and microRNAs (miRNAs). EBV was first identified in Burkitt lymphoma (BL) by the Epstein team in 1964 (8). EBV infection is highly prevalent in the general population, with nearly 90% of 10-year-old children in China being positive for serum EBV antibodies (5).

A prerequisite for the ability of EBV to cause disease is its successful invasion of host cells. EBV is transmitted primarily via saliva. Upon entering the oral cavity, it utilizes various viral envelope glycoproteins to bind to corresponding receptors, predominantly invading human B cells and epithelial cells in the pharynx and frequently involving T lymphocytes and NK cells-all of which express the CD21 receptor for EBV (9–11). EBV is abundant in the salivary glands and saliva of both patients and asymptomatic carriers. It establishes latent infection in human B lymphocytes, rendering the infected individual a lifelong virus carrier. Since the virus is present mainly in oral secretions, infected individuals, once they become sources of infection, can shed the virus continuously or intermittently through saliva and droplet transmission for weeks, months, or even years (12). Primary EBV infection is also possible via hematopoietic cell transplantation, solid organ transplantation, and blood transfusion (1, 13–15).

EBV can induce a variety of infectious diseases. Children may present with diverse clinical symptoms following primary EBV infection. However, owing to the immaturity and weak reactivity of the pediatric immune system, the body fails to mount an effective immune response against viral invasion postinfection, resulting in inconspicuous clinical manifestations that are easily overlooked. Most affected children may exhibit asymptomatic infection or mild pharyngitis and upper respiratory tract infection, whereas some may develop EBV-IM, the typical clinical manifestation of EBV infection (16–19). Although EBV-IM is mostly self-limiting, in the clinic, some children may have involvement of multiple organs and systems. In severe cases, it can progress to EBV-associated hemophagocytic syndrome, resulting in multiorgan/system dysfunction (20). Some children with EBV-IM may present with the typical clinical triad: fever, pharyngitis, and cervical lymphadenopathy. EBV can replicate in pharyngeal cells, causing cell destruction, which leads to symptoms of tonsillitis and pharyngitis, as well as enlargement of local involved lymph nodes (21). It can also enter the bloodstream and spread via viremia or infected B lymphocytes. Lymphocyte infiltration can cause immune-mediated injury, and children may develop hepatic injury, hepatitis, and hepatocellular injury (22). Massive activation of B lymphocytes can result in lymphadenopathy and hepatosplenomegaly (23).

The liver is the primary iron storage organ in the body, and iron metabolism is a key factor in maintaining normal hepatic function. Iron overload can induce lipid peroxidation via the Fenton reaction, exacerbating hepatocellular injury. Ferroptosis is emerging as a novel form of regulated cell death, which is closely associated with the progression of multiple liver diseases, such as cancer, liver fibrosis, and ischemia‒reperfusion injury (24, 25). Ferritin is a multifunctional protein that may play roles in proliferation, angiogenesis, antioxidation, and immunosuppression. Elevated ferritin levels reflect the degree of inflammation or tissue damage (26). TIBC can indicate the efficiency of iron utilization in the body (27). In this study, no significant differences in iron or ferritin levels were detected between the hepatic injury group and the non-hepatic injury group (*P* > 0.05). All children enrolled in this study were in the active phase of EBV-IM. Regardless of the presence of hepatic injury, both groups exhibited systemic lymphoproliferative inflammatory responses induced by EBV infection. Serum ferritin is not only an indicator of systemic iron stores, but also a classic acute-phase protein; the shared systemic inflammation in both groups may elevate ferritin levels, which largely masks the specific differences related to hepatic injury. Similarly, serum iron is susceptible to interference from multiple confounding factors such as inflammation and infection, which may explain the non-significant between-group differences. There was a statistically significant difference in TIBC levels between children in the hepatic injury group and the non-hepatic injury group. This result not only suggests that children in the hepatic injury group have lower systemic iron utilization efficiency, but also indicates that TIBC may be less susceptible to interference from the aforementioned confounding factors.

EBV DNA positivity is direct evidence of the presence of EBV. Existing studies have shown that the level of EBV DNA load can predict the occurrence, progression, and prognosis of diseases. It can be used to screen high-risk populations and guide the preventive treatment of organ transplant recipients, thereby reducing the risk of EBV-associated diseases (28). For instance, high EBV DNA levels in peripheral blood mononuclear cells have been proven to be associated with the development of post-transplant lymphoproliferative disorders (29); in patients with nasopharyngeal carcinoma, plasma EBV DNA has been confirmed to reflect the disease stage, treatment efficacy, and prognosis (30); natural killer/T-cell lymphoma patients with positive plasma EBV DNA detection before and after treatment may have a higher risk of recurrence (31); and the severity of EBV-associated hepatitis has been shown to be related to high EBV DNA levels (32, 33). Reports in the literature suggest that, compared with the EBV DNA load, lymphocyte dysfunction is more severe in patients with hepatic injury, and the occurrence of hepatic injury may not be directly associated with the EBV DNA load (34). However, other studies have shown that an EBV DNA concentration >500.00 × 10^4^ copies/mL is a risk factor for EBV-IM-related hepatic injury. Studies have also shown that blood viral load is positively correlated with the severity of the disease (21). This contradicts the conclusions of the aforementioned reports (35). In this study, a whole blood EBV DNA load exceeding 1 × 10^5^ copies/mL was identified as one of the risk factors for hepatic injury in children with EBV-IM. Nevertheless, there are few relevant reports on the relationship between the EBV DNA load and EBV-IM-related hepatic injury, necessitating further verification through additional studies.

This study analyzed potential factors affecting hepatic function in patients with EBV-IM. However, the objective of this study was not to predict the occurrence of hepatic injury; instead, based on existing data, it analyzed the correlative patterns of laboratory characteristics among EBV-IM patients with established hepatic injury (especially moderate to severe cases), so as to provide a reference for clinically identifying the associated indicators in such patients.The novelty of this study lies in the inclusion of analyses of serum iron, ferritin, and total iron-binding capacity alongside traditional indicators, as well as the introduction of the NLR and PLR on the basis of routine blood tests. The TIBC was an independent risk factor for hepatic injury caused by pediatric EBV-IM, whereas the PLR was a protective factor. Additionally, a whole blood EBV DNA load exceeding 1 × 10^5^ copies/mL was identified as a risk factor for hepatic injury in children with EBV-IM.

This study has several limitations. First, it is a cross-sectional, single-center retrospective study. On the one hand, it cannot establish a causal relationship; on the other hand, it relies on historical data, which may introduce bias and lead to deviations in the results. Second, the study cases were only collected from Jilin Province, resulting in limited geographical representativeness, and the results may not reflect the overall situation across China. Third, specific data on hepatosplenomegaly in children were not specifically recorded or analyzed, and were only indicated by positive events. Meanwhile, the definition of hepatic injury in this study focused more on moderate to severe cases [total bilirubin ≥4 mg/dL (68 μmol/L) or alanine aminotransferase (ALT) exceeding twice the age-appropriate upper normal limit], leading to incomplete coverage of patients with mild abnormalities in hepatic function indicators.

Currently, large-sample, multicenter prospective studies are still needed to further analyze the risk factors for hepatic injury caused by pediatric EBV-IM.

## Data Availability

The datasets presented in this article are not readily available due to privacy or ethical restrictions. Requests to access the datasets should be directed to the corresponding author.
